# Renal Vein Thrombosis on Point-of-care Ultrasound in the Emergency Department: A Case Report

**DOI:** 10.5811/cpcem.2021.9.53714

**Published:** 2022-01-24

**Authors:** Michelle Haimowitz, Laura K. Gonzalez

**Affiliations:** Maimonides Medical Center, Department of Emergency Medicine, Brooklyn, New York

**Keywords:** case report, renal vein thrombosis, point-of-care ultrasound, renal ultrasound

## Abstract

**Introduction:**

This case report of renal vein thrombosis found on emergency bedside ultrasound illustrates the expanding role of point-of-care ultrasound (POCUS) in rapidly identifying rare renal pathologies.

**Case Report:**

A 16-year-old female with a complex medical history presenting with right-sided abdominal pain and tenderness was found to have significant renal POCUS findings consistent with renal vein thrombosis.

**Conclusion:**

In the medically complex patient with nonspecific chief complaints, it can be challenging to rapidly narrow a broad differential diagnosis. Point-of-care ultrasound has proven to be an extremely useful tool for this purpose. As emergency physicians become more proficient in the use of ultrasonography, it is likely that POCUS will be used with increasing frequency to identify additional pathology outside its traditional applications.

## INTRODUCTION

During the last few decades, point-of-care ultrasound (POCUS) has become an important tool in the emergency department (ED) for rapid and accurate diagnosis of many conditions. It enables emergency physicians to answer specific clinical questions in a timely manner leading to improved patient care. Historically, renal ultrasonography (RUS) has been used in the ED to assess the presence or absence of hydronephrosis which is most commonly associated with obstructive uropathy or to identify urinary retention. With the widespread use of renal POCUS by trained emergency physicians other pathologies have been identified, contributing to the diagnosis of a broader range of disease than ever before. The following case illustrates the role of renal POCUS in identifying unusual renal pathology and expediting care in a medically complex patient.

## CASE REPORT

A 16-year-old female with a past medical history significant for well controlled human immunodeficiency virus (HIV) infection, idiopathic thrombocytopenic purpura (ITP), menorrhagia, anemia, thyroid disease, and bilateral cholesteotomas with conductive hearing loss presented to the ED with severe acute right-sided abdominal pain and non-bilious, non-bloody emesis for eight hours. Of note, she had undergone a complicated right tympanomastoidectomy two days prior. The patient endorsed constipation but was passing flatus; she denied fevers, chills, cough, shortness of breath, diarrhea, hematochezia, melena, urinary complaints, or headaches. Her current medications included eltrombopag, abacivir-lamivudine, dolutegravir, levothyroxine, cephalexin, and ferrous sulfate. She denied sexual activity and had no history of tobacco, alcohol, or drug use.

The patient’s vital signs on presentation were temperature 97.8° Fahrenheit, heart rate 87 beats per minute, respiratory rate 18 breaths per minute, blood pressure 112/78 millimeters (mm) of mercury, and oxygen saturation of 100% on room air. On exam, she was noted to be in moderate distress secondary to pain. Her right ear was covered by a large dressing; there was no scleral icterus, and she had moist mucous membranes. Her neck was supple and her lungs were clear. Heart sounds were regular with normal rate; perfusion was normal. There was notable right upper and lower quadrant tenderness with guarding but no masses, rebound, or costovertebral tenderness. The patient was alert and appropriately responsive with a grossly normal neurological exam. No rash, petechiae, or bruising were noted. While in the ED, the patient experienced two episodes of gross hematuria.

**Image 1:**
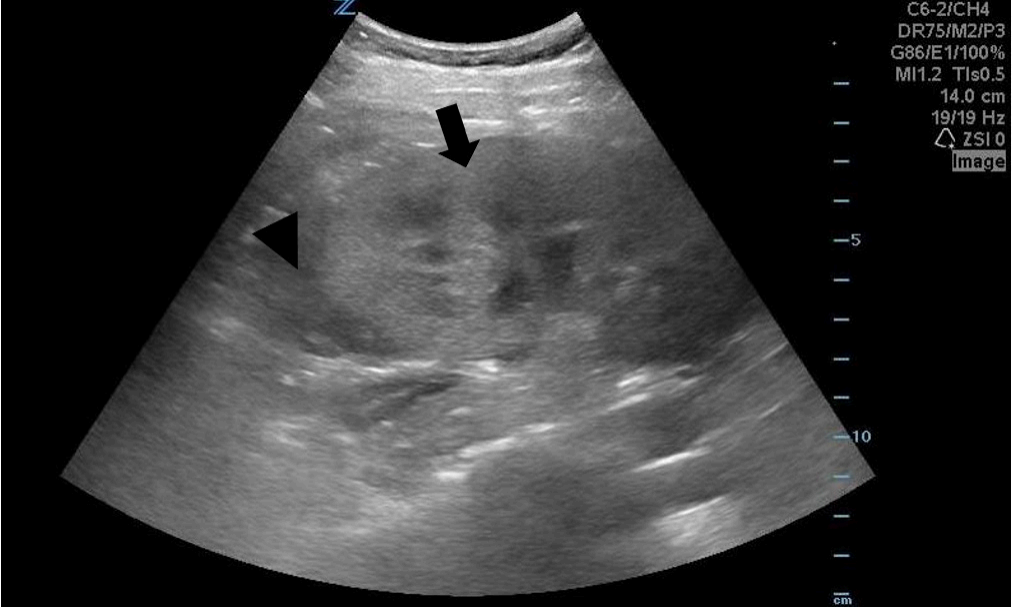
Point-of-care ultrasound of the right kidney seen in the longitudinal plane with curvilinear probe showing loss of corticomedullary differentiation (arrow) and surrounding free fluid (arrowhead).

**Image 2:**
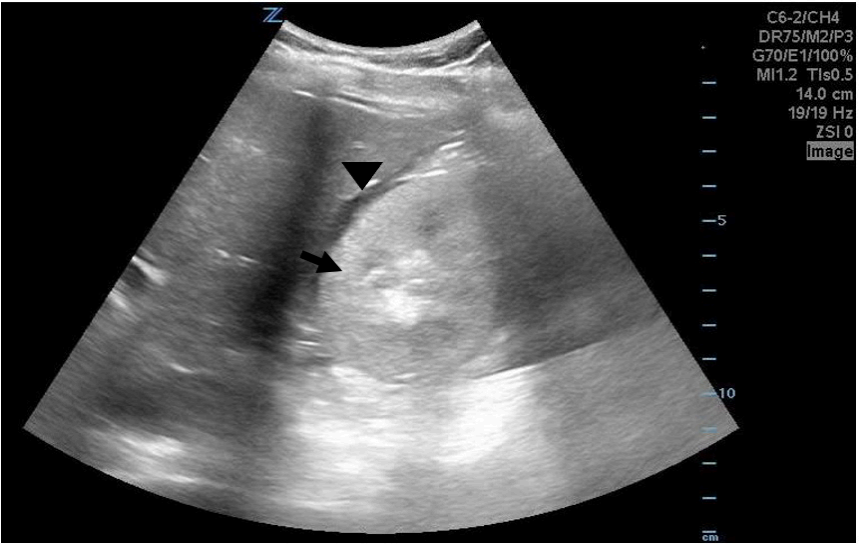
Point-of-care ultrasound of the right kidney seen in the transverse plane with curvilinear probe showing an enlarged and hyperechoic kidney (arrow) with surrounding free fluid (arrowhead).

Given the history and exam, a broad differential was considered including biliary tract disease, appendicitis, ovarian pathology, ureterolithiasis, urinary tract infection, and small bowel obstruction. Labs were notable for a white blood cell count of 21.8 thousands per cubic millimeter (K/uL) (reference range: 4.8–10.8 K/uL) with 94% neutrophils (50–70%), a hemoglobin of 10.1 grams per deciliter (g/dL) (12.0–16.0 g/dL), and platelets of 98 K/uL (150–400 K/uL); normal electrolytes, renal function, liver function, and coagulation tests; and a urinalysis with proteinuria, ketonuria, hemoglobinuria, urobilinogen, and small leukocyte esterase. (Images 1, 2 and Video) The patient was given intravenous (IV) hydration and pain medications after which a renal POCUS was performed. Sonographic findings were remarkable for mild right hydronephrosis, an enlarged kidney with surrounding free fluid, and a diffusely hyperechoic renal cortex with decreased corticomedullary differentiation. Although the underlying cause of these findings was uncertain, it was clear that the source of her abdominal pain was renal. She was sent for computed tomography (CT) with IV contrast showing an “edematous right kidney with markedly delayed enhancement, prominent surrounding inflammation and fluid, and fullness of the right renal vein with hypodensity extending into the inferior vena cava suspicious for renal vein thrombosis (RVT).”

**CPC-EM Capsule**

What do we already know about this clinical entity?

*Acute renal vein thrombosis (RVT) is a rare condition often seen in the setting of hypercoagulable states. Computerized tomography angiography and magnetic resonance angiography are usually used to diagnose this condition*.

What makes this presentation of disease reportable?

*Although sonographic findings have been reported previously in radiology literature, point-of-care ultrasound (POCUS) findings of RVT have never been described in the emergency department literature*.

What is the major learning point?

*As the use of POCUS increases, physicians will identify findings of both common and uncommon diseases; this in turn will facilitate more refined and expeditious patient care*.

How might this improve emergency medicine practice?

*We hope that presenting this case will encourage emergency physicians to use POCUS to narrow differential diagnoses and expedite patient care*.

Urology and vascular surgery were immediately consulted for thrombosis of the right renal vein with concern for ischemia of the kidney; otolaryngology (ENT) was also consulted given the patient’s recent surgery. Vascular Surgery recommended anticoagulation if deemed safe given her recent surgery; ENT agreed with anticoagulation, and treatment with enoxaparin was commenced. Eltrombopag was stopped given the associated risk of hypercoagulability. The patient was given ceftriaxone in light of the abnormal urinalysis and possible urinary tract infection, and she was admitted to the pediatric service for further evaluation and management.

While inpatient, additional workup revealed a pulmonary embolism in the left lung base. The patient developed fever and was treated for superimposed pneumonia. Additionally, a heart murmur prompted an echocardiogram, which was negative for endocarditis. Additional consults included infectious disease, hematology/oncology, cardiology, and nephrology. She was discharged home but returned with multiple pulmonary emboli a week later. She underwent another hospital stay during which an extensive thrombophilia workup was negative. It is thought that the most likely cause of her disease process was a combination of underlying HIV and ITP, use of eltrombopag, and relative immobility secondary to surgery.

## DISCUSSION

Point-of-care ultrasound has revolutionized the way emergency physicians evaluate patients at bedside. Whereas previously the exam was limited to visualization, auscultation, palpation and percussion, POCUS now allows physicians to directly visualize the underlying disease. Many abdominal pathologies can be successfully and rapidly identified at the bedside including gallbladder disease, small bowel obstruction, appendicitis, intussusception, hydronephrosis, and more. Additional benefits of POCUS include the avoidance of radiation, the ability to assess function, and the ability to do serial exams. The renal system is particularly well-visualized on ultrasound, which has a long and trusted role in diagnosis of renal pathology.

The primary application of renal POCUS has been the diagnosis of hydronephrosis, which is most often due to ureterolithiasis. The gold standard for urinary tract stones has been computed tomography (CT); however, there are many reasons CT may not be optimal including the risk of radiation, the relatively higher cost, a lack of availability, and an associated increased length of stay. In the hands of trained emergency physicians, renal POCUS has been shown to be a useful tool to identify the presence or absence of hydronephrosis, which may alleviate the necessity for further imaging.^1^

A randomized, multicenter controlled trial of patients with suspected urinary tract stones that compared ultrasound to CT found no difference in high-risk diagnoses with complications, serious adverse events, pain scores, hospital admissions, or ED readmissions in the two groups.^2^ Furthermore, although ultrasound is less sensitive for renal colic, its use over CT did not lead to worse patient-centered outcomes. Leo et al. further investigated the use of ultrasound for urinary tract stones and found that the degree of hydronephrosis could effectively rule out the presence of stones greater than five mm, which more often require surgical intervention. While there were higher rates of return to the ED within 30 days, this was found to be due to pain rather than serious adverse events or complications, further supporting the idea that ultrasound is a safe alternative to CT.^3^

In cases of suspected renal colic where there is no or only mild hydronephrosis on POCUS, studies have shown that patients are significantly less likely to have larger stones that require surgical intervention. In such cases, Goertz et al. argues that the use of POCUS and urinalysis is sufficient to diagnose renal colic. By avoiding CT in these patients, its use would be decreased by 73% while only missing 9% of patients with calculi greater than 5 mm. Additionally, none of these missed patients had stones greater than 10 mm, and thus all would have been appropriate for a trial of medical management.^4^

While the presence or absence of hydronephrosis is the primary clinical question the emergency physician must answer, clinicians using POCUS have become more adept at identifying additional renal pathologies. There are multiple case reports of other diseases identified on renal POCUS including renal trauma,^5^ renal cell carcinoma,^6^ urinomas,^7^ emphysematous pyelonephritis,^8^ pyonephrosis,^9^ and xanthrogranulomatous pyelonephritis.^10^ Furthermore, its routine use in some other conditions has been advocated. Chen et al. conducted a retrospective review of patients diagnosed with acute pyelonephritis (APN) and found that emergency renal ultrasonography identified pathology in 60.9% of complicated APN. (“Complicated APN” is defined as admission longer than 14 days, admission to intensive care unit, and need for invasive treatments.) More significantly, however, was that 34.3% of these patients were found to have significant sonographic abnormalities that led to a change in management including surgical interventions such as percutaneous nephrostomy, abscess aspiration, ureteroscopic stone manipulation, lithotripsy, or nephrectomy.^11^

In the case of our patient, renal POCUS was used to rapidly identify the kidney as the source of her acute presentation; given her complicated medical history and highly abnormal renal POCUS, further imaging was pursued. Acute RVT is a rare condition most often seen in the settings of dehydration, trauma, infection, nephrotic syndrome, or hypercoagulable states.^12^ It can present similarly to renal colic with flank pain and hematuria and thus would often prompt renal POCUS in the ED. While the gold standard for diagnosis of RVT is selective renal venography, both CT angiography and magnetic resonance imaging have gained favor as they are less invasive.^13^ Very few studies have investigated the use of ultrasound to diagnose RVT, and although sonographic findings have been reported previously in the radiology literature, to our knowledge they have never been described in the ED literature. Classic sonographic signs of RVT include abnormal echogenicity, which can be increased or decreased depending on the age of insult^14^ and “loss of corticomedullary differentiation in addition to renal enlargement.”^15^ Color Doppler can also be used to assess flow in the renal vein.^14^ These descriptions are consistent with the ultrasound findings seen in this patient, further supporting the final diagnosis made by the emergency physician using POCUS in conjunction with CT.

## CONCLUSION

Point-of-care ultrasound is an extremely useful tool available to emergency physicians, and its use is associated with decreased radiation exposure, reduction of healthcare costs, improvement in time to diagnosis, and better patient outcomes. Additionally, it has contributed to the identification of less common pathologies leading to life-saving interventions. The full spectrum of its utility has yet to be revealed. In the case reported here, the patient had an extensive medical history and presented with nonspecific abdominal pain leading to a broad differential diagnosis. Use of POCUS allowed the emergency care team to rapidly identify renal pathology and to tailor further workup accordingly, thus optimizing care for this very complicated pediatric patient with renal vein thrombosis.

## Supplementary Information

Video Point-of-care ultrasound of the right kidney seen in the longitudinal plane with curvilinear probe showing loss of corticomedullary differentiation (black arrow) and surrounding free fluid (white arrow).
